# Conceptualizing Lennox–Gastaut Syndrome as a Secondary Network Epilepsy

**DOI:** 10.3389/fneur.2014.00225

**Published:** 2014-10-30

**Authors:** John S. Archer, Aaron E. L. Warren, Graeme D. Jackson, David F. Abbott

**Affiliations:** ^1^Department of Medicine, Austin Health, The University of Melbourne, Heidelberg, VIC, Australia; ^2^Florey Institute of Neuroscience and Mental Health, Heidelberg, VIC, Australia; ^3^Department Neurology, Austin Health, Heidelberg, VIC, Australia

**Keywords:** Lennox–Gastaut syndrome, generalized epilepsy, tonic seizure, EEG–fMRI, default-mode network, attention network, paroxysmal fast activity, slow spike and wave

## Abstract

Lennox–Gastaut Syndrome (LGS) is a category of severe, disabling epilepsy, characterized by frequent, treatment-resistant seizures, and cognitive impairment. Electroencephalography (EEG) shows characteristic generalized epileptic activity that is similar in those with lesional, genetic, or unknown causes, suggesting a common underlying mechanism. The condition typically begins in young children, leaving many severely disabled with recurring seizures throughout their adult life. Scalp EEG of the tonic seizures of LGS is characterized by a diffuse high-voltage slow transient evolving into generalized low-voltage fast activity, likely reflecting sustained fast neuronal firing over a wide cortical area. The typical interictal discharges (runs of slow spike-and-wave and bursts of generalized paroxysmal fast activity) also have a “generalized” electrical field, suggesting widespread cortical involvement. Recent brain mapping studies have begun to reveal which cortical and subcortical regions are active during these “generalized” discharges. In this critical review, we examine findings from neuroimaging studies of LGS and place these in the context of the electrical and clinical features of the syndrome. We suggest that LGS can be conceptualized as “secondary network epilepsy,” where the epileptic activity is expressed through large-scale brain networks, particularly the attention and default-mode networks. Cortical lesions, when present, appear to chronically interact with these networks to produce network instability rather than triggering each individual epileptic discharge. LGS can be considered as “secondary” network epilepsy because the epileptic manifestations of the disorder reflect the networks being driven, rather than the specific initiating process. In this review, we begin with a summation of the clinical manifestations of LGS and what this has revealed about the underlying etiology of the condition. We then undertake a systematic review of the functional neuroimaging literature in LGS, which leads us to conclude that LGS can best be conceptualized as “secondary network epilepsy.”

## Lennox–Gastaut syndrome – Definition and Clinical Features

Lennox–Gastaut Syndrome (LGS) is a severe epilepsy phenotype, usually beginning in childhood, and commonly associated with intellectual disability. Onset of LGS is typically before the age of 8 years (1–3), with peak onset age between 3 and 5 years (4). Once established, 80% of LGS patients will continue to have seizures into adulthood (5, 6). Individual patients may have a variety of genetic abnormalities or cortical lesions (7), and in a significant proportion of patients, perhaps 25% (8, 9), the underlying cause is unknown.

**Figure 1:**
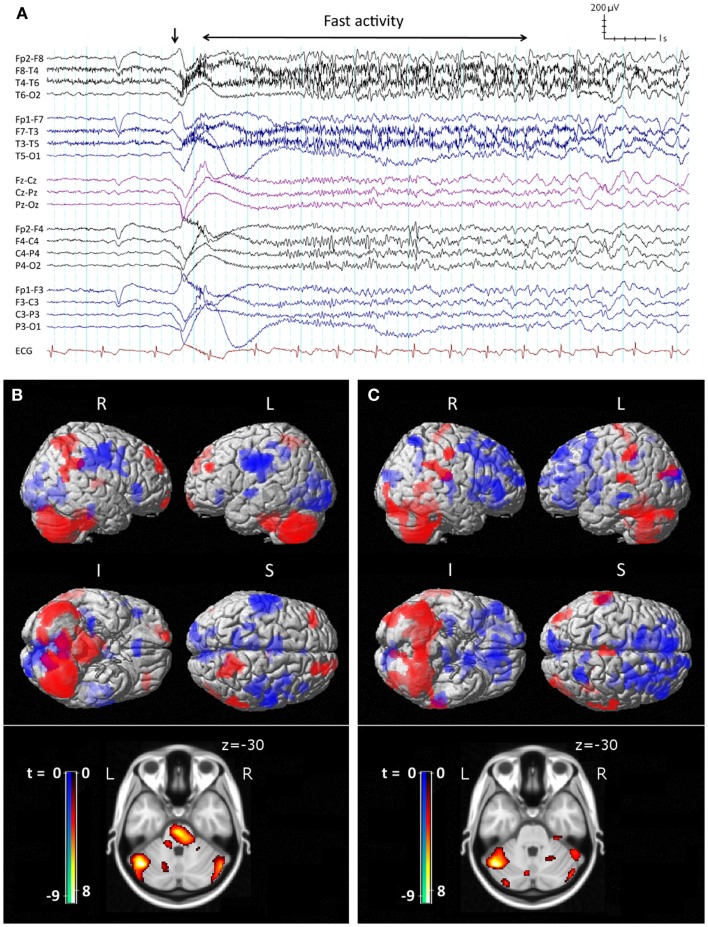
**Ictal EEG features and peri-ictal SPECT of tonic seizures in LGS**. **(A)** Clinical onset of seizure corresponds with a high-voltage slow transient (vertical arrow) followed by apparent diffuse attenuation, evolving into low-voltage fast activity (LVFA) and later a run of slow spike-and-wave mixed with notched delta. **(B)** Early radiotracer injection (<10 s after offset of LVFA) and subsequent SPECT shows an early pattern of increased (red) cerebral blood flow in frontal and parietal “attention” areas, pons, and cerebellum, and decreased (blue) CBF in primary cortical areas. **(C)** Late radiotracer injection (>10 s after offset of LVFA) and subsequent SPECT shows an evolution toward a pattern of increased CBF over lateral parietal cortex and cerebellum, and decreased CBF bi-frontally, while the pons is no longer involved. **(B,C)** Top: surface renderings displayed at *p* < 0.02 (uncorrected), extent *k* > 125 voxels. Below: overlay onto axial slice of MNI T1 152 average brain displayed at *p* < 0.05 [cluster-corrected for family-wise error (FWE)]. R = right, L = left, I = inferior, S = superior. Adapted and re-printed with permission from Intusoma and colleagues (13).

The core features of LGS were described by Henri Gastaut in 1966 (10). Patients may have a variety of seizure types, often with multiple daily attacks, but tonic seizures, which cause patients to suddenly and unpredictably stiffen and drop to the ground, are a key diagnostic feature (11). On electroencephalography (EEG), tonic seizures are characterized by a diffuse high-voltage slow wave followed by generalized low-voltage fast activity (LVFA) (Figure 1A), likely reflecting sustained fast neuronal firing over a wide cortical area (12). The interictal EEG shows frequent runs of pseudo-rhythmic 1.5–2.5 Hz diffuse slow spike-and-wave (SSW), and intermittent bursts of generalized paroxysmal fast activity (GPFA), particularly in sleep (4). The electrical features of GPFA show similarity to the LVFA of tonic seizures, suggesting that they probably recruit similar brain networks.

Although LGS is relatively uncommon [0.24–0.28 per 1,000 births; (14, 15)], the persistent nature of seizures results in a relatively high prevalence, estimated at 1–10% of all children with epilepsy (8, 16–21), and 3–17% of patients with epilepsy and intellectual disability (22–24). LGS patients are not uncommon in epilepsy clinics.

### Lennox–Gastaut “phenotype”

Patients with some, but not all, the features of LGS, were previously classified as having “secondary generalized epilepsy” (6, 25, 26). This term was removed from the 2010 International League Against Epilepsy (ILAE) updated classification of the epilepsies (11), as it was felt the diagnostic category had become an unhelpful “dumping ground” for poorly defined cases of severe epilepsy. It is clear that many recent advances in understanding disease mechanisms in epilepsy have come from genetic discoveries, derived from careful electroclinical phenotyping (27, 28). Unfortunately, in clinical practice, this has meant patients who manifest most of the electrical features of LGS (tonic seizures, SSW, and GPFA), but who might have an older than usual age of onset, minimal EEG background slowing, or mild intellectual disability, are no longer easily classified. We have begun using the term “Lennox–Gastaut Phenotype” (LGP) to describe these patients (29), as we believe the similarities in electroclinical expression likely reflect similarities in the neural networks being driven by epileptic activity.

### Epileptic encephalopathy

Lennox–Gastaut Syndrome is classified as one of the epileptic encephalopathies (11), as it seems likely that the epileptic process pervasively inhibits cognition and cognitive development. Patients with LGS frequently show cognitive regression around the time of diagnosis, while established LGS is almost always associated with moderate to severe cognitive impairment. Twenty to sixty percent of patients show intellectual disability at the time of diagnosis, increasing to 75–95% within 5 years of the syndrome’s onset (4). Fifty-five percent of LGS patients have an IQ under 50 (30), and impairment is often global. On continuous performance tasks, children and adolescents with LGS show impaired information processing with marked slowing of reaction times to cognitive and motor stimuli (31). Behavioral and psychiatric disturbances are frequent in LGS, compounding the burden of care. Common problems include aggressiveness, hyperactivity, and autistic traits (32–38). Long-term outcomes are typically very poor, with the majority of patients remaining under home-care or institutionalization (2, 39), and some needing to wear a helmet to prevent seizure-related head and face injuries (40).

Cognitive impairment in LGS appears related to the age of onset and persistence of seizures. An earlier age of seizure onset (<5 years) has been associated with more severe cognitive impairment, while patients who develop LGS later in life (>9 years) follow a more favorable cognitive course (3, 30, 32, 41–43). In a group of patients with normal mental development before the onset of LGS, Ohtsuka (44) found that 95.7% (22/23) of patients with persistent seizures showed cognitive impairment after a follow-up period of at least 5 years compared with 12.5% (1/8) of patients who had been seizure free for at least 1 year.

If seizures remain poorly controlled, there appears to be progressive cognitive impairment over time. Oguni (1) followed 72 patients for a mean of 17 years and found a decrease of at least 15 IQ points from onset of diagnosis to end of follow-up in around 80% of patients with LGS. In contrast, there are a number of case reports of improved cognitive trajectory in patients with LGS due to a lesion, who become seizure free following resective surgery (45, 46).

### Variable causes, common electroclinical features

No single pathophysiology underlies the development of LGS (25), although the age-dependent expression implies that there is something about the immature brain that renders it susceptible to development of the LGS phenotype (47). Approximately 10–30% of patients have an epileptogenic abnormality visible on structural MRI (3, 48), with focal, multifocal, or diffuse structural abnormalities described. Etiologies include focal cortical dysplasia, perinatal anoxia, ischemic stroke, intracranial hemorrhage, and encephalitis (7, 49). A variety of genetic factors, particularly *de novo* mutations, have been implicated in some patients (7, 50). However, approximately 25% of patients with LGS (8, 9) have no obvious structural brain abnormalities and no confirmed genetic abnormalities. These cases may be considered LGS of unknown cause (11). It is notable that the electroclinical features of tonic seizures and interictal discharges in LGS are remarkably similar whether or not there is a causative lesion, and independent of lesion location or pathology. Conversely, the same etiology may lead to LGS or a more benign epilepsy phenotype. For example, tuberous sclerosis is a condition, in which inherited or spontaneous mutations of the TSC1 or TSC2 gene lead to a failure of inhibition of the mTOR (mammalian target of rapamycin) pathway, causing abnormal cell proliferation. In this condition, defects in the same molecular pathway, and at times the same genetic abnormality, may produce epileptic spasms, an LGS phenotype, or focal epilepsy (51, 52). Hence, there are factors other than the specific molecular mechanism that determine whether a patient will express the LGS phenotype.

### Potentially reversible

**Figure 2:**
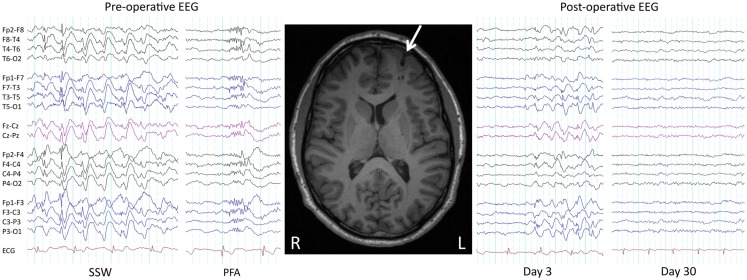
**Pre- and post-operative EEG**. Pre- and post-operative EEG in a 38-year-old male with LGS, a lesion, and intractable seizures since childhood. Prior to resection of a left frontal cortical dysplasia (arrowed), the patient suffered daily seizures. Pre-operative interictal EEG showed bursts of slow spike-and-wave (SSW) and generalized paroxysmal fast activity (GPFA). Day 3 post-operative EEG showed persistence of SSW, while day 30 EEG showed complete normalization, consistent with a winding down of the epileptic process. The patient is 2 years seizure free, consistent with LGS being potentially reversible. Re-printed with permission from Archer and colleagues (29).

Seizures and developmental delay are not necessarily permanent in LGS. With regards to seizures, as early as 1979 it was shown that surgical removal of a parietotemporal neoplasm in a child with LGS led to a complete remission of seizures and SSW patterns on EEG (53). We recently showed similar improvements in three patients who had their lesions removed (29) (Figure 2), consistent with other reports of seizure freedom following focal or lobar resections in LGS patients with parietal, frontal, temporal, and hypothalamic lesions on MRI (45, 46, 54–62). Following successful epilepsy surgery, some LGS patients show an initial persistence of seizures or “generalized” epileptic discharges, which subsequently resolve (“winding down”; Figure 2) (29, 45, 46, 54, 56). This demonstrates that although lesions can cause the LGS phenotype, at least in some patients the lesions themselves are not triggering each individual epileptic discharge (“secondary bi-synchrony”). It suggests instead that lesions are interacting with key networks to create an unstable mode of network behavior (29). Once the destabilizing influence is removed, in this case the cortical lesion, cerebral networks are able to gradually return to a more stable (non-epileptic) state.

In addition to reductions in seizure frequency and normalization of EEG abnormalities, there are several reports of post-operative cognitive gains (45, 46, 54, 55, 57–60), supporting the notion that intellectual deterioration may in part be due to seizures and interictal discharges (63, 64). For example, Liu (59) performed comprehensive pre- and post-operative neuropsychological assessment in 15 patients with LGS who underwent single-lobe/lesionectomy or multi-lobe resection, and found a significant mean IQ increase from 56.1 to 67.4 after surgery. These benefits become less certain as duration of LGS prior to surgery increases (59, 65), a trend found in other severe childhood epilepsies (66–69). Hence, there appears to be a time window in which the epileptic brain is both vulnerable to irreversible cognitive decline and amenable to treatments that restore normal development.

## Involvement of subcortical structures

### Thalamus

The generalized nature of epileptic discharges and seizures has led many to postulate that the thalamus may be a key initiator of epileptic activity in LGS. Recordings from the thalamus during generalized epileptic discharges of LGS confirm that the thalamus is involved (70, 71). EEG–functional magnetic resonance imaging (fMRI) studies have shown thalamic involvement during SSW (29, 72, 73) and generalized spike-and-wave (74, 75). High-frequency electrical deep brain stimulation (DBS) of the thalamic centromedian nucleus has been reported to reduce generalized seizures by 80% in a group of 13 LGS patients (76). However, given that cortical lesions can cause LGS, and their removal can lead to abolition of the epileptic process, it seems likely in this case that the thalamus is probably acting as a synchronizer and amplifier, rather than initiator.

### Pons

The pons appears involved in tonic seizures. Direct electrical stimulation of pons in animals reproduces posturing similar to a tonic seizure, with predominant axial muscle involvement (77). Auditory stimulation of the brainstem in a rat model of generalized epilepsy causes animals to have convulsive attacks with electrophysiological evidence of excessive brainstem firing, but no evidence of cortical involvement (78). We have shown increased blood flow in the pons during tonic seizures in humans, consistent with increased pontine neuronal activity (13). However, as noted above, cortical lesions can cause LGS, and their removal can lead to abolition of the epileptic process. Hence, although the pons is involved in seizure expression, it does not appear to be the initiator of epileptic activity and seizures (79).

## A Network Disorder

The shared electroclinical and cognitive features of LGS suggest that common cerebral networks are involved. Epilepsy is increasingly being recognized as a disorder of cerebral networks (29, 72, 74, 80–84). The electroclinical features of an epilepsy syndrome can be considered as reflecting the specific cerebral networks being recruited. In this context, a neural network comprises anatomically and functionally connected cortical and subcortical brain structures, where activity in any one part of the network may affect activity in all the others (80). Network-based considerations of epilepsy are useful and clinically relevant because they can help explain seizure semiology can suggest which cerebral networks may be dysfunctional in the interictal state, and can help guide medical and surgical management directions. For example, the diagnosis of temporal lobe epilepsy (TLE), reflecting seizures predominantly expressed in the limbic system, makes sense of the memory, olfactory, and other symptoms the patient may experience during an “aura.” It permits interpretation of ictal features, including spread patterns. It suggests particular imaging and genetic studies directed at epilepsy involving this region, while leaving open the idea that seizure activity could have started elsewhere (e.g., occipital lobe) but be maximally expressed through the temporal lobe. Finally, the label of TLE helps interpretation of memory deficits, which are associated with dysfunction of this particular network. The neuroimaging evidence for network involvement in LGS is reviewed below.

## Epilepsy Networks of LGS: A Systematic Review of the Functional Neuroimaging Literature

In this section, we review the functional neuroimaging studies in LGS, in particular, positron emission tomography (PET), interictal and peri-ictal single-photon-emission computed tomography (SPECT), and combined electroencephalography (EEG) and functional magnetic resonance imaging (EEG–fMRI).

### Search strategy

A literature search in the bibliographic database PubMed (1982 to April 2014) was undertaken. Search terms were restricted to articles’ titles and abstracts. A combination of the following search terms was used: LGS AND PET OR positron OR SPECT OR photon OR fMRI OR EEG–fMRI OR neuroimaging. Furthermore, we examined each article’s reference list and used Google to search for websites that might provide additional references. This effort resulted in 95 citations that were selected for review. Included articles were limited to studies reporting primary data; review articles were read but are excluded here. A total of 70 citations were excluded as irrelevant, with 25 remaining for review.

### Positron emission tomography

The most common radio-ligand is fluoro-2-deoxy-d-glucose (FDG-PET), which images glucose uptake, to display average cerebral metabolism over the course of the image acquisition, usually 30–60 min in duration (85). Several interictal PET studies with small numbers of LGS patients have shown unilateral focal or multifocal hypometabolic abnormalities, predominantly in frontal and temporal regions, that tend to correlate with structural abnormalities observed on structural imaging or epileptic foci determined by EEG (86–89). Others have observed more diffuse abnormalities, including generalized bilateral hypometabolism, most prominent fronto-temporally (90). Some patients show normal cerebral glucose metabolism (91). The variability in these results was recapitulated in a larger series of 15 children with LGS (92), where four major metabolic subtypes were identified: unilateral focal hypometabolism in frontal or temporal regions; unilateral diffuse hypometabolism; bilateral diffuse hypometabolism; and normal metabolic patterns. Ferrie (93) aimed to establish whether PET would reveal focal abnormalities in a group of LGS patients who had no localizing features evident on clinical examination, EEG, or high resolution MRI. Using asymmetry indices for patients’ own homologous cortical regions to detect metabolic defects, no focal abnormalities were found in patients with *de novo* LGS, while LGS cases following West syndrome more commonly showed unilateral focal hypometabolism in temporal, frontal, or parietal regions. Repeat PET performed 1 year later in a subset of patients with focal abnormalities showed that hypometabolic defects were stable over time (94). In a further semi-quantitative analysis comparing metabolic rates in LGS to age-matched controls, Ferrie (95) observed widespread, generalized hypometabolism in cortical and thalamic regions in LGS patients with and without previously reported focal abnormalities (93). The degree of hypometabolism in the frontal lobes was later reported to be inversely correlated with measures of patients’ adaptive behavior (96).

Taken together, these results agree with the clinical impression that LGS is a disorder of heterogeneous etiologies. However, they add further evidence that, in some cases at least, generalized epileptic activity in LGS may be caused by focal cortical abnormalities. This notion is supported by more recent uses of PET in the identification of metabolic defects in LGS patients who undergo resective surgery and subsequently show seizure improvement (57, 58, 60). An additional observation across these studies is that in patients who do show aberrant metabolic activity, whether focal, multifocal, or diffuse, the abnormality appears largely confined to association cortex (involving frontal, temporal, and parietal lobes), typically sparing primary cortical areas (e.g., primary visual and motor cortex). This pattern of common cerebral network involvement has been observed in other functional neuroimaging modalities, which are discussed below.

### Single-photon-emission computed tomography

**Figure 3:**
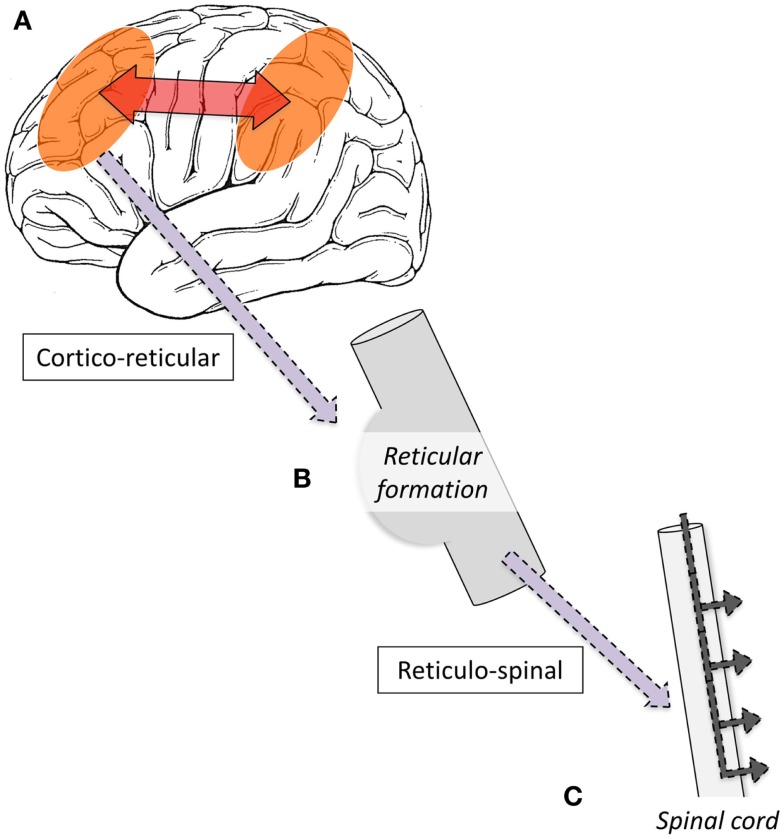
**Schematic illustration of proposed mechanism of tonic seizures in LGS is shown**. **(A)** Epileptiform activity initiated in cortex, and rapidly amplified within intrinsic attention and default-mode networks. **(B)** Epileptiform activity projects via cortico-reticular pathway – Brodmann area 6 (premotor cortex) to ponto-medullary reticular formation (105, 106). **(C)** Epileptiform activity projects via the reticulo-spinal pathway to motor neurons innervating proximal muscles at multiple levels (107).

Single-photon-emission computed tomography is able to image regional cerebral blood flow (CBF) to identify brain regions that are active during a seizure (97, 98). To date, very few studies have been performed in LGS patients. A small number of case reports have found diffuse foci of reduced CBF in frontal, temporal, or parietal regions (99–103); however, their interpretation is limited because studies were only performed interictally, making it difficult to differentiate normal from epileptogenic tissue (104). To address this gap in the literature, we recently performed a voxel-wise comparison of ictal and interictal SPECT in a group of 10 scan pairs from 7 LGS patients who were studied during video EEG-confirmed tonic seizures (13). Five patients had focal structural abnormalities on MRI. Across the whole group, tonic seizures were associated with increased CBF in the lateral parietal lobe and cerebellum, and reduced CBF bilaterally in frontal and occipital regions. The evolution of CBF changes was also explored by examining patient subgroups who were injected with a radiotracer early (<10 s) or late (>10 s) after the offset of EEG LVFA (Figure 1). The early injection group showed increased CBF in the pons, cerebellum, and bilateral fronto-parietal regions, and reduced CBF in primary cortical areas, including pericentral and occipital cortex. The late injection group showed an evolution of this pattern toward increased CBF over lateral parietal cortex and the cerebellum, and reduced CBF frontally. Despite some of these patients having a focal cortical lesion in different locations, we observed a common pattern of early association cortex involvement and reduced activity in primary cortical areas. We postulated that tonic seizures in LGS reflect activity in a corticopontine pathway, arising in a network of bilateral frontal and parietal association cortices before projecting via cortico-reticular pathways to the pons, and from there via reticulo-spinal pathways to spinal motor neurons (Figure 3).

### EEG–fMRI

An understanding of the importance of cerebral networks in epilepsy has been driven by insights gained through combined EEG and functional MRI (EEG–fMRI) studies, including key publications from our laboratory (29, 72, 74, 75, 108–114). Recording low-voltage scalp EEG signals in the MR environment poses a number of challenges (115–117), but these can be largely overcome (118, 119).

Electroencephalography–functional magnetic resonance imaging utilizes the blood–oxygen-level-dependent (BOLD) response (120) to visualize activity changes associated with epileptiform discharges across the whole brain. Functional MRI activity represents summed local field potentials across time [fMRI volume acquisition time (TR) is typically 2–3 s] and space (voxel size is typically 3–5 mm^3^) (121). Hence, EEG–fMRI provides an overview of cerebral network behavior during epileptic discharges. Indeed, because fMRI is sensitive to brain activity that is not necessarily hyper-synchronized, it can do more than simply map the brain regions active at the time of the spike; it can also map brain activity time-locked to but preceding the EEG spike (113, 122), thus providing a more complete picture of the brain networks associated with epileptic discharges.

A relatively small number of EEG–fMRI papers have examined LGS. One study of spike-and-wave activity in 16 subjects with “secondary generalized epilepsy” who were scanned at 1.5 T showed thalamic activation in addition to widespread cortical changes that included variable activation and deactivation in frontal and parietal regions (123). Similar results were observed in two of these patients who were studied again at 3 T using simultaneous EEG with BOLD and arterial spin label (ASL) fMRI (124). BOLD activation and deactivation during spike-and-wave observed in frontal and parietal regions corresponded, respectively, with CBF increases and decreases recorded with ASL. A group analysis of 11 children with LGS, with EEG–fMRI performed under chloral hydrate sedation, found BOLD increases in the thalamus and brainstem (73). These changes were found on an analysis that combined all discharges, including SSW and “polyspike” discharges, potentially diluting the differential effects of SSW and GPFA on cortical activity.

**Figure 4:**
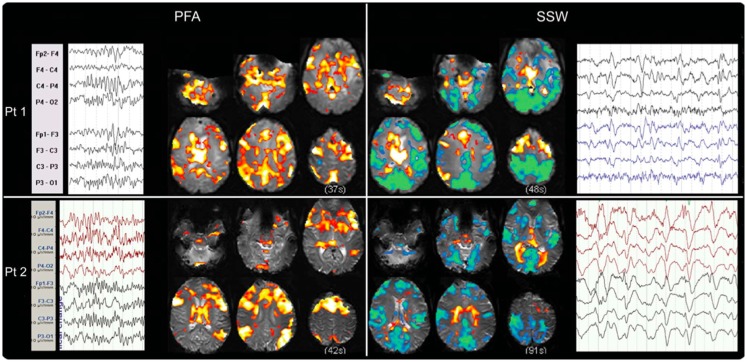
**Electroencephalography–functional magnetic resonance imaging of generalized paroxysmal fast activity (GPFA) and slow spike-and-wave (SSW) in individual LGS patients is shown**. In individual patients, GPFA and SSW produce different blood–oxygen-level-dependent (BOLD) response patterns. GPFA shows increased BOLD in diffuse association network regions, as well as brainstem, basal ganglia, and thalamus. SSW shows a different pattern, with decreased BOLD signal in primary cortical areas. The number of events in seconds, at the bottom of each panel, is the sum of the length of all individual epileptiform events recorded during the EEG for each patient. Pt, patient. Re-printed with permission from Pillay and colleagues (72).

**Figure 5:**
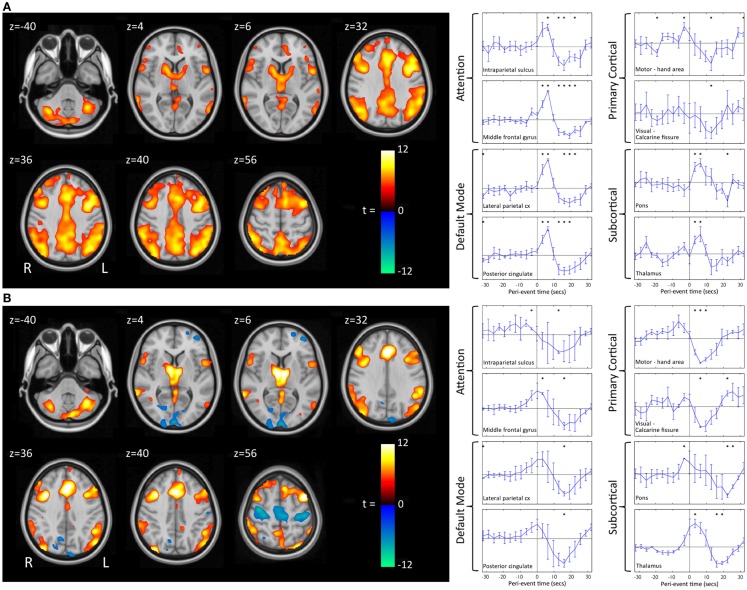
**Group-level EEG–fMRI activation maps and peri-event BOLD signal time-courses in LGS patients with epileptogenic lesions in different cortical locations are shown**. **(A)** Generalized paroxysmal fast activity (GPFA). *Left*: fixed-effects whole-brain group EEG–fMRI analysis in six patients with cortical lesions in different locations showing co-activation of two normally anti-correlated cognitive systems in diffuse association cortex: the attention and default-mode networks. Activations are displayed as two-tailed *t*-statistics thresholded at *p* < 0.05 (corrected for FWE) and overlaid on axial slices of the MNI T1 152 average brain. *Right*: random-effects peri-event time-course analysis showing GPFA group mean BOLD signal change from regions of interest. Time-courses are displayed in 3.2 s time-bins, from 32 s before to 32 s after event onset (indicated by vertical line). Error bars indicate standard errors. Asterisks indicate time-bins of significant mean BOLD signal change (two-tailed single sample *t*-tests, *p* < 0.05, uncorrected). Time-course analysis confirms simultaneous BOLD signal increases in frontal and parietal association cortical areas, thalamus, and pons, and reduced signal in primary cortical areas. **(B)** Slow spike-and-wave (SSW). *Left*: fixed-effects whole-brain group EEG–fMRI analysis in three subjects with cortical lesions in different locations showing mixed increased and decreased BOLD signal, including activation in thalamus and lateral frontal and parietal areas, and deactivation in primary cortex including pericentral and occipital regions. Activations are displayed as per **(A)**. *Right*: random-effects peri-event time-course analysis showing SSW group mean BOLD signal change from regions of interest, as displayed in **(A)**. Time-course analysis shows a complex set of activity changes that are only partially captured by the whole-brain maps. Attention and default-mode networks are being driven simultaneously, but with a steady “pre-spike” increase in activity followed by a decrease in signal at the time of scalp-detected SSW. Primary cortical regions show signal decreases. Adapted and re-printed with permission from Archer and colleagues (29).

We have recently shown that GPFA and SSW, the two pathognomonic interictal discharges of LGS, are associated with quite different changes in neuronal activity (29, 72). GPFA is associated with diffuse association network activation (Figure 4), consistent with the GPFA EEG signature of widespread fast activity. Association cortex contains two dominant cognitive systems: the attention network, which modulates focused attention to task across a range of cognitive domains; and the default-mode network (DMN), that engages during quiet reflection, reminiscing, and internal thinking. Neural activity in these two networks is normally anti-correlated, consistent with their diametrically opposed cognitive functions (125–128). Epileptiform activity in LGS appears to be associated with a highly unusual pattern of co-activation of attention networks and the DMN. Furthermore, there is a very similar pattern of network activation in LGS whether or not there is an underlying epileptogenic lesion, and independent of lesion location (Figure 5), supporting our hypothesis that the shared electroclinical features of LGS reflect underlying similarities in the recruited brain networks.

Slow spike-and-wave also appears to simultaneously recruit the attention and DMNs, but with a more complex pattern (Figure 4). SSW shows a steady upward drift of activity for more than 6 s prior to scalp-detected activity, followed by an abrupt fall in activity with the appearance of SSW on the scalp (Figure 5). The curious phenomenon of “pre-spike” fMRI activity changes has been observed in generalized spike-and-wave of genetic generalized epilepsy (74, 129–131), and may reflect the need of the brain to be in a specific state for spike-and-wave discharges to occur. The shape of the hemodynamic changes around the time of SSW is a poor fit for the canonical hemodynamic response function (HRF) that is typically used in event-related analyses to generate maps of activity changes (132). This may explain the variability seen with our EEG–fMRI maps of SSW. EEG–fMRI studies of epileptic discharges in other epilepsy syndromes have also observed that BOLD responses to discharges show differences to the canonical HRF (113, 114, 133–135).

We and others have observed reduced activity in primary cortical regions during interictal discharges (GPFA and SSW; Figures 4 and 5) (29, 73). This is consistent with our observation of reduced blood flow in sensorimotor cortex during tonic seizures (13). Hence, it appears that epileptic activity in LGS is not predominantly expressed through primary cortical regions. This suggests that an alternate pathway generates the axial predominant movements of tonic seizures, perhaps cortico-reticular pathways (105, 106), driving the pontine reticular formation, with outflow via reticulo-spinal projections, which innervate predominately axial muscles at multiple spinal levels (107) (Figure 3).

## Conclusion

Patients with LGS have a similar electroclinical phenotype, despite varying etiologies, consistent with a common underlying mechanism. The EEG features suggest that there is widespread cortical recruitment during epileptic activity. Functional neuroimaging has confirmed that epileptic activity in LGS recruits widespread areas of association cortex (diffuse association network activity), and spares primary cortical regions. Hence, LGS appears to be a network epilepsy, where the epileptic discharges and seizures reflect abnormal neuronal firing within intrinsic cognitive brain networks, specifically the attention and DMNs. Furthermore, epileptic activity in LGS appears to be characterized by a fundamental breakdown in normal brain network behavior, with co-activation of attention networks and the DMN. However, it is not yet clear whether it is the attention network, the DMN, or both that are key to the LGS phenotype.

The epileptic process in LGS appears to be initiated from the cortex. Cortical lesions can cause LGS, and their removal can abolish seizures. Some patients show “winding down” of interictal discharges following removal of an epileptogenic lesion. This strongly suggests that cortical lesions, when present, chronically interact with these networks to produce network instability rather than triggering each individual epileptic discharge. Presumably, a wide range of molecular and neuronal mechanisms could produce a similar pattern of network instability. In patients without an obvious cortical lesion, therapies that seek to reduce network instability, such as “generalized” anti-convulsants, are likely to be beneficial (4). Preliminary evidence suggests that thalamic DBS may also be beneficial (76), possibly by modulating network excitability. Although the epileptic process is driven from the cortex, it appears that tonic seizures are expressed through the reticular formation of the pons. We propose that when epileptic activity in the cognitive networks reaches a particular threshold, it triggers cortico-reticular pathways, which connect premotor cortex (Brodmann area 6) to the pontine reticular formation. Trunkal predominant movement is likely generated via reticulo-spinal pathways, which innervate axial muscles at multiple levels. These primitive pathways are normally responsible for postural control and orienting behavior, such as turning to visual, auditory, or tactile stimuli (105, 106), but in LGS are being driven by epileptic outflow from the cortex.

Lennox–Gastaut syndrome can be conceptualized as secondary network epilepsy, where the epileptic discharges and seizures reflect epileptic activity being amplified through intrinsic cognitive brain networks. The epileptic features of LGS reflect activity in these networks, rather than the specific lesional, genetic, or other cause. We believe that the label of “secondary network epilepsy” is useful as it captures and explains the key electroclinical features, including tonic seizures, SSW, and GPFA. The label allows initial management decisions to be made, including consideration of “generalized” drug therapies, while acting as a reminder to continue to search for specific underlying causes. Finally, the label reminds us that the process is potentially reversible, if an underlying treatable cause such as a lesion can be identified early.

## Conflict of Interest Statement

The authors declare that the research was conducted in the absence of any commercial or financial relationships that could be construed as a potential conflict of interest.
